# Between Silence and Stigma: Stakeholder Perspectives on Sociocultural Resistance to Human Papillomavirus Vaccination in Lebanon

**DOI:** 10.7759/cureus.88080

**Published:** 2025-07-16

**Authors:** Joumana L Merhi, David M Atallah, Salim M Adib, Ibrahim R Bou-Orm

**Affiliations:** 1 Higher Institute of Public Health, Faculty of Medicine, Saint Joseph University of Beirut, Beirut, LBN; 2 Faculty of Medicine, Department of Gynecology and Obstetrics, Saint Joseph University of Beirut, Beirut, LBN; 3 Department of Gynecologic Surgery, Hotel-Dieu de France University Hospital, Beirut, LBN; 4 Department of Epidemiology & Population Health, American University of Beirut, Beirut, LBN; 5 Department of International Public Health, Liverpool School of Tropical Medicine, Liverpool, GBR

**Keywords:** culture-centered approach, health communication, hpv vaccination, lebanon, professional discourse, public trust, sexuality, sociocultural resistance, stigma, vaccine hesitancy

## Abstract

Background

Despite the role of human papillomavirus (HPV) vaccination in preventing cervical cancer, uptake in Lebanon remains minimal. While previous research has primarily focused on public knowledge and attitudes as well as policy and economic evaluations of a national adoption, limited attention has been given to how sociocultural norms, stigma, and silence contribute to resistance. This study addresses this gap by exploring the perspectives of national stakeholders.

Methods

Twenty-two semi-structured interviews were conducted with government health officials, medical experts, representatives of scientific societies, public health and communication experts, civil society actors, and staff from UN agencies and health financing institutions. Data were thematically analyzed following Braun and Clarke’s method and interpreted through the lens of the Culture-Centered Approach (CCA).

Results

Three interrelated themes emerged: (1) widespread silence and misinformation about HPV, reinforced by gaps in education and media engagement; (2) cultural taboos and gendered moral expectations complicating vaccination acceptability; and (3) fragmented professional discourse and eroding public trust in health actors. These dynamics interact to reinforce sociocultural resistance.

Conclusions

Resistance to HPV vaccination in Lebanon is rooted not only in misinformation and limited awareness but also in silence, stigma, fragmented professional discourse, and public mistrust. Addressing these barriers requires culturally resonant strategies guided by the CCA, engaging trusted social actors, and fostering inclusive dialogue to build cultural acceptance and public trust.

## Introduction

Resistance to vaccination due to sociocultural factors poses a significant barrier to public health, particularly for interventions linked to reproductive health and sexuality. Although human papillomavirus (HPV) vaccination is a proven cancer prevention measure, its uptake in Lebanon remains minimal. At the time of the study, the vaccine was available only through private clinics and pharmacies, at full out-of-pocket cost, and was not included in the national immunization program [1,2].

Several studies have examined the determinants of low HPV vaccination acceptance in Lebanon. These include affordability, weak visibility in health communication and school-based education, insufficient physician recommendations, and widespread misinformation. Quantitative research has consistently documented limited awareness among adolescents, mothers, and even medical students, with a strong link between knowledge and vaccination intentions [2-6]. Parental hesitation is further shaped by safety concerns and the absence of strong pediatrician recommendations [7], while physicians highlight systemic constraints such as cost, lack of coverage, and inconsistent clinical practices [1]. Some studies also point to cultural taboos and gendered norms as underlying factors in vaccine hesitancy, particularly regarding adolescent sexuality and reproductive health [8]. However, these findings are primarily drawn from survey-based studies and focus largely on parents, youth, and individual healthcare providers, rather than on institutional or professional framings.

To date, the only known qualitative research in Lebanon remains a doctoral dissertation focused on student perceptions [9]. No peer-reviewed studies have qualitatively explored sociocultural resistance from institutional or professional perspectives.

This study builds on this body of work by examining HPV vaccination resistance through the lens of national-level stakeholders, including government health officials, medical experts, representatives of scientific societies, public health and communication professionals, civil society actors, and staff from UN agencies and health financing institutions. By qualitatively exploring how professional and institutional actors interpret stigma, silence, and moral discomfort, the study offers a novel contribution to the literature, providing insights relevant to other conservative and middle-income countries where science, sexuality, and public trust intersect. To our knowledge, this is the first study to investigate how sociocultural dynamics are understood by those shaping national discourse and policy around HPV vaccination in Lebanon.

Stakeholder perspectives are interpreted through the lens of the Culture-Centered Approach (CCA), which emphasizes the role of culture in shaping health meanings and communication [10,11]. While the CCA addresses the interplay of structure, culture, and agency, this analysis focuses specifically on the cultural dimension, examining how silence, stigma, and gendered moral expectations shape vaccine resistance. By exploring both spoken and unspoken dynamics across clinical, professional, and media settings, the study surfaces the communicative practices and symbolic norms that shape public discourse and influence HPV vaccination acceptance.

This study aims to explore sociocultural resistance to HPV vaccination in Lebanon from the perspective of national-level institutional stakeholders, with a focus on the cultural dynamics of silence, stigma, and communicative norms, interpreted through the CCA.

## Materials and methods

Study design and purpose

This paper presents a focused, theory-informed sub-analysis of a broader qualitative study on HPV vaccination policymaking in Lebanon. While the parent study explored multiple dimensions, including structural, financial, and political factors, this analysis isolates sociocultural dynamics and interprets them through the lens of the CCA, emphasizing how meanings, taboos, and silences shape health communication. While the narrative flow of participant accounts is preserved in the presentation of findings, the analysis follows a thematic design, as outlined by Braun and Clarke [12].

Although the CCA addresses the interplay of structure, culture, and agency, this analysis foregrounds the cultural dimension. Constructs such as institutional silence and professional ambiguity, while also reflecting structural and agentic dimensions, are treated here as culturally embedded phenomena that shape and are shaped by shared communicative practices and symbolic norms.

This sub-analysis focuses exclusively on cultural dynamics. While cost-related barriers were examined in the parent study, they are intentionally excluded from this article to maintain thematic and theoretical coherence. No results from other analytic frameworks are duplicated here; this article addresses a distinct research question within the broader study.

Reflexivity and positionality are integral to this approach, requiring researchers to critically examine their own assumptions and interpretive power [10,11,13]. The lead researcher (J.M.) is a Lebanese public health professional and a PhD candidate in Public Health. She conducted all interviews and led the analysis. Her dual role as insider and academic interpreter informed both data generation and thematic interpretation, while collaborative review by co-authors helped strengthen analytical reflexivity and reduce interpretive bias.

Participant selection

We employed purposive sampling to identify 22 national-level stakeholders from diverse sectors relevant to HPV vaccination discourse and policy in Lebanon. Participants were selected based on their institutional roles and relevance to the topic. They included public health and health communication experts, civil society actors, representatives of scientific societies and professional orders, national advisory committee members, government health officials, medical experts (e.g., pediatricians, gynecologists, infectious disease specialists), and staff from UN agencies and health financing institutions. Inclusion criteria were (1) familiarity with HPV vaccination discourse or delivery in Lebanon; (2) involvement in health-related policymaking or public communication; and (3) willingness to participate in a semi-structured interview.

Data collection

Data were collected between February and May 2024 as part of a broader qualitative study examining the political, economic, institutional, and sociocultural factors influencing the introduction of HPV vaccination into Lebanon’s national immunization calendar. A semi-structured interview guide was used to explore multiple dimensions, including existing cervical cancer prevention policies, structural and financial constraints, stakeholder roles, power dynamics, and decision-making processes.

This paper presents a focused sub-analysis of that dataset, specifically examining sociocultural influences on HPV vaccination. Relevant questions from the guide addressed public perceptions of HPV vaccination, cultural taboos, stigma, moral norms, and physician communication practices. Interviews were conducted either in person or via secure online platforms and lasted 30-60 minutes. All interviews were audio-recorded with participant consent, except one, which was documented through detailed notes at the participant’s request.

Data analysis

Interviews were transcribed, anonymized, and analyzed thematically using Braun and Clarke’s (2006) method [12]. Coding was conducted inductively using Dedoose software (SocioCultural Research Consultants, LLC, Los Angeles, CA). The analysis focused on sociocultural constructs including stigma, silence, moral framing, and trust. The first author led coding and theme development, with collaborative review to ensure consistency and analytic credibility. Peer debriefing and iterative coding strengthened trustworthiness.

Following thematic analysis, the CCA was applied as an interpretive framework to contextualize the emergent sociocultural themes. While the CCA did not inform data collection or initial coding, it enabled deeper engagement with the cultural meanings embedded in stakeholder narratives, particularly around communicative silences, symbolic norms, and moral expectations.

The excerpts presented in this article were selected from a broader interview dataset based on their salience to the cultural constructs under investigation, including silence, stigma, moral framing, and trust. Thematic saturation was reached for the sociocultural themes, with no new codes emerging in the final interviews.

This analysis reflects both the perspectives of national-level stakeholders and the interpretive lens of the research team. We acknowledge that the focus on institutional actors provides an expert, policy-facing view rather than a grassroots perspective. Findings may also be shaped by the positionalities of the researchers as public health professionals.

Ethical considerations

Ethical approval was obtained from the Ethics Committee of Hôtel-Dieu de France, Saint Joseph University (reference number CEHDF2323). Informed consent was obtained from all participants, and all data were anonymized to ensure confidentiality.

## Results

Findings are presented through a culture-centered lens, highlighting how shared norms, moral framings, and selective silences shape the social environment surrounding HPV vaccination. Three interrelated themes structure sociocultural resistance in Lebanon: (1) knowledge gaps and misinformation, (2) cultural taboos and gendered moral expectations, and (3) fragmented medical voices and public mistrust. Resistance manifests as silence, confusion, discomfort, and distrust, rooted in prevailing social norms and inconsistent messaging across families, schools, media, and healthcare.

Theme 1: Knowledge gaps and misinformation

Stakeholders highlighted that public awareness of HPV in Lebanon remains limited. They described a landscape where misinformation thrives and where open conversations about the virus are notably scarce, not just within communities, but also in schools and healthcare settings. Even among those who have heard of HPV, many hold inaccurate beliefs about its health risks and the value of vaccination. This gap is made worse by the lack of structured public education. A government health official explained, “There are people who never heard about the vaccine and do not know that it exists, and they are not following up; others have few misconceptions which might be deleterious for the introduction; and others know about it but do not know about its impact” (Participant 1).

These gaps are especially acute in less privileged communities, where access to reliable information is limited by socioeconomic status and exposure to proactive health professionals. A professional order representative observed, “the knowledge is limited to the people that are in the domain… but the ‘tabaka sha’biyye’ (grassroots populations), no, they don’t have knowledge about it” (Participant 7). A UN agency expert similarly noted, “Some of the people that have more exposure and more education… are more prone to vaccinate their kids” (Participant 4).

Within the health sector, awareness is inconsistent, with some physicians relying on pharmaceutical representatives rather than scientific literature. A public health expert noted, “The primary source of information is the ‘prospecteur’ (medical representative), not reading articles; few of them read scientific articles, but most of the physicians in Lebanon get their information from the pharmaceutical firms” (Participant 15). A UN agency expert added, “I cannot honestly say that all physicians work based on a scientific way, evidence-based way, especially those who practice in remote areas and in dispensaries” (Participant 4). Despite these challenges, some positive changes are emerging, such as school-based initiatives and NGO-led campaigns, though these remain scattered and largely inaccessible to the general public.

Theme 2: Cultural taboos and gendered moral expectations

Cultural sensitivities surrounding sexuality remain a central barrier to HPV vaccine acceptance. Unlike childhood vaccinations, the HPV vaccine often causes discomfort due to its association with adolescent sexual activity. Many families reject the vaccine, believing it to be unnecessary based on assumptions about premarital abstinence. A scientific society representative explained, “A lot of families, when we talked about offering the vaccines to their children, they used to say: ‘No this is a vaccine for the West… our girls don’t go out until they get married’” (Participant 10).

Health professionals may also internalize these norms, resulting in inconsistent advocacy for HPV vaccination. As a medical expert explained, “Unfortunately, we still see physicians who are not encouraging the HPV (vaccine) because they mix their cultural beliefs and traditional background with their practice” (Participant 18). A scientific society representative echoed this concern, noting, “I have been arguing with some of my colleagues… that was always the reasoning: ‘Why we should give it… they’re not going to be sexually exposed’” (Participant 10).

These moral expectations reinforce silence rather than informed dialogue, with discussions about HPV often avoided in both clinical and educational settings. The vaccine’s association with reproductive organs and its role in preventing a sexually transmitted infection render it more socially sensitive than other cancer-prevention tools. As a health communication expert observed, “Under the appearance of a liberal country, we are still governed by communities; there are many taboos” (Participant 14). The same expert elaborated, “For AIDS, there was also a taboo. AIDS was perceived as something you get if you are sexually active… So, some people were cynically saying, ‘they deserve it’… So, you have social and religious taboos because it is associated with sexual activity” (Participant 14).

In response to these sociocultural challenges, many stakeholders emphasized the need to reframe the HPV vaccine primarily as a cancer prevention intervention, rather than as protection against a sexually transmitted infection. “Bringing this vaccine as a cancer prevention vaccine rather than an STI prevention vaccine will be very critical,” explained one UN agency expert (Participant 6). Several participants echoed this view, suggesting that decoupling vaccination from moral judgment could help reduce resistance. As a government health official noted, “You don’t have to ask your daughter if she’s having sex… You can just vaccinate her, and that’s it” (Participant 3).

Others highlighted the importance of engaging trusted intermediaries, particularly within Lebanon’s religious, educational, and civil society sectors, to support broader acceptance. “Once the decision is made, then the implementation needs to take the NGOs, religious leaders, community-based organizations, media; how to implement this educational population awareness,” observed a national advisory committee representative (Participant 12). A government health official added, “You can start with very visible schools to show… and everyone would want to follow” (Participant 3).

Together, these perspectives suggest that careful messaging and the involvement of respected community actors could help overcome prevailing taboos and improve public understanding and acceptance of HPV vaccination.

Theme 3: Fragmented medical voices and public mistrust

Stakeholders described variability in physicians’ engagement with HPV vaccination, shaped by differences in training, areas of specialization, and exposure to continuing medical education. As a government health official explained, “Even the physicians… it is a mixed population, it is not all the same. Those who work in this field, like gynecologists and infection diseases, might know more about it” (Participant 1). Reflecting on their own specialization, the same official noted, “It depends on education and awareness… For me… I don’t know much about it… we were not trained on it, because we don’t deal with it” (Participant 1).

Tensions between medical specialties were also seen as a source of confusion and fragmentation. A national advisory committee representative recalled, “We had competition amongst physicians. Pediatricians who wanted to vaccinate earlier… Ob-gyn said, ‘we will do it before she gets married, we will do it at the age of 18,’ just to shift population from pediatric to ob-gyn… it was a disaster… very harmful” (Participant 12). Interviewees described this lack of coordination as contributing to inconsistent recommendations regarding the appropriate timing for vaccination.

In addition to interpersonal dynamics, several participants pointed to the absence of unified institutional guidance and the visible role of pharmaceutical companies in promoting the vaccine. While some interviewees acknowledged the value of these awareness efforts, others expressed concern over the reliance on industry-driven messaging in the absence of neutral, evidence-based frameworks.

These professional and institutional gaps were viewed as contributing to a broader context of public mistrust. As a public health official remarked, “The public they are like a sponge that absorbs everything according to how we present it to them” (Participant 2). Participants noted that inconsistent advice from clinicians, combined with limited official communication, often left individuals confused or hesitant.

Trust in public health institutions emerged as a recurrent theme. A UN agency representative observed, “A challenge very specific to Lebanon… is related to the trust in the public sector” (Participant 6). Even when vaccines are offered at low or no cost through government channels, skepticism around safety, quality, and intent was said to persist.

Despite these challenges, some interviewees identified areas of progress. Civil society groups and international agencies were seen as playing a more active role in raising awareness, and some participants reported growing public interest in the vaccine. However, most emphasized that rebuilding trust would require credible, transparent communication and more inclusive outreach strategies.

Participants also called for broader engagement across the health workforce. As a professional order representative argued, “The beginning should start with the healthcare providers, to provide them enough knowledge regarding this vaccine… and not to be confined to physicians only… we should have all doctors, midwives, nurses” (Participant 7). Expanding training and coordination across professional roles was viewed as essential to delivering coherent messages and strengthening public confidence.

Table 1 summarizes the key findings from each theme, highlighting the distinct yet interrelated sociocultural dynamics shaping HPV vaccine resistance in Lebanon.

**Table 1:** Key sociocultural themes influencing resistance to HPV vaccination in Lebanon HPV, human papillomavirus.

Theme	Interpretive Summary
1. Knowledge Gaps and Misinformation	Public awareness of HPV and its vaccine is limited and uneven, especially among underserved communities. Misinformation persists across all settings, including among physicians, partly due to reliance on pharmaceutical sources and the lack of structured public education.
2. Cultural Taboos and Gendered Moral Expectations	Cultural taboos surrounding adolescent sexuality and gendered moral expectations influence both public and provider attitudes. The vaccine is often perceived as unnecessary or inappropriate, reinforcing silence and limiting open dialogue.
3. Fragmented Medical Voices and Public Mistrust	Gaps in physician knowledge, training, and clinical engagement contribute to inconsistent guidance. In the absence of cohesive institutional messaging, public trust in the health system remains limited, reinforcing confusion and skepticism around the vaccine.

## Discussion

This study explored sociocultural resistance to HPV vaccination in Lebanon from the perspective of national institutional stakeholders. While previous research in Lebanon has identified limited awareness, cost concerns, and provider hesitation as barriers to uptake, sociocultural dynamics such as silence, stigma, and moral discomfort have received limited empirical attention. By foregrounding these cultural dimensions, the study responds to a key gap in the literature and contributes a deeper understanding of how symbolic norms, communicative practices, and professional discourse shape vaccine resistance. Specifically, participants described how culturally shaped silences around sexuality, moral framings of adolescent behavior, and inconsistent professional discourse contribute to hesitancy and resistance - themes that emerged inductively and were interpreted through the CCA.

Sociocultural resistance to HPV vaccination in Lebanon cannot be reduced to knowledge gaps or conservative values. Instead, it emerges from a complex interplay of selective silence, moral ambivalence, and culturally embedded communication norms. In this context, silence is not merely the absence of dialogue but an active, patterned practice that shapes who speaks, what is discussed, and in which settings. These silences extend across families, schools, clinical environments, and media, often reflecting an avoidance of discomfort or controversy around sexuality and prevention [10].

Persistent gaps in public and professional awareness are sustained by these entrenched silences. The absence of structured school-based education, national data, and consistent media engagement allows misinformation to thrive. Providers frequently fail to recommend HPV vaccination or align with international guidelines, contributing to inconsistent discourse and missed opportunities for prevention. Similar dynamics are observed in other middle-income countries, where provider hesitancy and cultural taboos constrain vaccine uptake [2,14].

Cultural discomfort with adolescent sexuality and gendered moral expectations strongly shape HPV vaccine perceptions. In Lebanon, vaccination is sometimes interpreted as tacit approval of premarital sexual activity, particularly for girls. Discourses around virginity, religious propriety, and parental control reinforce these views and restrict open dialogue. This resonates with regional findings showing how sexuality-related stigma impedes reproductive health communication [15]. According to the CCA, such taboos persist precisely because they remain unspoken, structuring what is considered legitimate to say, to whom, and in what form [10]. These communicative norms shape both public discussion and clinical behavior, limiting advocacy and fueling uncertainty.

Physician engagement with HPV vaccination remains fragmented, influenced by professional identity, clinical training, and broader sociocultural context. Ambiguity about who should recommend the vaccine - pediatricians, gynecologists, or general practitioners - further fuels this inconsistency. Reliance on pharmaceutical promotion, limited continuing education, and discomfort discussing sexual health all contribute to contradictory messages. These findings align with broader literature showing that unclear professional roles and incentives hinder vaccine advocacy [16,17]. These patterns also reflect underlying epistemic hierarchies in which institutional and commercial actors shape what knowledge is legitimized or disseminated [10].

Low trust in Lebanon’s public health institutions compounds these dynamics. Even if vaccines were offered through public channels, skepticism would remain around their safety, quality, and intent. This mistrust mirrors broader patterns observed in fragile health systems, where inconsistent communication and perceived inequities erode institutional legitimacy [18,19]. In this context, institutional silence around sexual and reproductive health is interpreted as disengagement, reinforcing hesitancy and resistance.

While this analysis centers the cultural dimension of the CCA, the findings also reveal how cultural, structural, and agentic processes interlink. Silences, stigmas, and fragmented messaging are culturally embedded yet reinforced by institutional gaps and professional hierarchies. At the same time, stakeholder calls for improved messaging, inclusive dialogue, and stronger provider engagement reflect latent agency - a potential for cultural transformation that is grounded in lived experience and communicative action. Breaking these silences and reducing stigma requires deliberate, participatory strategies that empower both institutions and communities to reshape the cultural conditions surrounding HPV vaccination.

**Figure 1:**
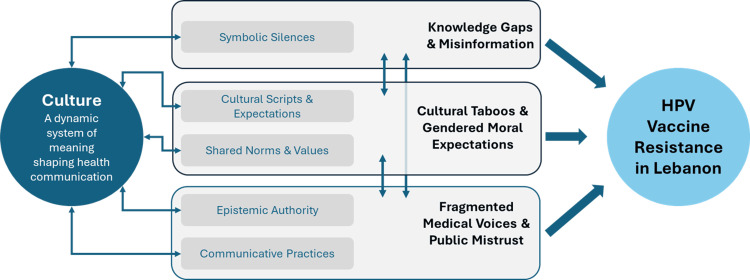
Conceptual framework illustrating how cultural processes, silences, and communicative norms contribute to HPV vaccine resistance in Lebanon. Interpretively adapted from the Culture-Centered Approach. HPV, human papillomavirus.

To visually contextualize these sociocultural dynamics, Figure 1 and Table 2 map each emergent theme onto relevant cultural constructs, illustrating how silence, stigma, and moral discourse converge to shape vaccine resistance. While Table 2 foregrounds cultural constructs, many also reflect structural constraints and latent agency, consistent with the integrative nature of the CCA framework. These visuals were developed interpretively following inductive coding to align the thematic findings with the CCA’s theoretical lens.

**Table 2:** Summary of cultural constructs by theme and CCA dimension Note: Constructs are not exclusive to individual themes and may intersect across thematic boundaries. CCA, Culture-Centered Approach.

Theme	Cultural Processes	Cultural Constructs	Primary CCA Dimension
1. Knowledge Gaps & Misinformation	Communicative silence; Misinformation; Access inequity	Symbolic silences; Structural inequity	Culture; Structure
2. Cultural Taboos & Gendered Moral Expectations	Stigma; Gender norms; Moral framing; Selective silence	Cultural scripts and expectations; Shared norms and values	Culture
3. Fragmented Medical Voices & Public Mistrust	Professional ambiguity; Institutional silence; Epistemic hierarchy; Mistrust	Epistemic authority; Communicative practices; Social response	Culture; Structure; Agency

Together, Figure 1 and Table 2 reinforce the central insight that sociocultural resistance in Lebanon is shaped as much by what is left unsaid as by what is explicitly communicated. They surface the often-invisible logics that govern public health discourse, revealing how silence functions both as a social constraint and as a potential site for transformation.

Aligned with the participatory ethos of the CCA, future interventions should be co-designed with community members. Centering lived experience in both the development and delivery of communication strategies is critical to overcoming stigma and rebuilding trust [11,20]. This includes equipping health professionals with unified, culturally sensitive messaging, engaging educators and religious leaders as dialogue facilitators, and fostering inclusive communication spaces. Grounded in participants’ calls for clarity, credibility, and engagement, such participatory approaches are key to achieving sustained HPV vaccine acceptance.

Limitations

This study has several limitations. First, it reflects the perspectives of national-level stakeholders and does not include direct input from community members, parents, adolescents, or religious leaders, offering an institutional rather than grassroots perspective. Second, although participants came from a variety of sectors, their views may not fully capture the diversity of cultural attitudes and experiences across Lebanon’s different communities. Third, as with all qualitative research, findings are context-specific and not meant to be statistically generalizable; however, they offer insights that may be relevant to other settings characterized by social conservatism, institutional fragility, or the coexistence of multiple health beliefs and practices. Finally, some participants may have shaped their responses to align with socially acceptable views, especially on sensitive topics such as sexuality, religion, or trust in government, introducing the possibility of response bias. In addition, participants’ institutional affiliations may have influenced how freely they expressed dissenting or critical views, potentially introducing a degree of self-censorship, particularly on politically or socially sensitive issues.

## Conclusions

This study shows that sociocultural resistance to HPV vaccination in Lebanon is shaped less by overt opposition and more by silence, stigma, and communicative ambiguity. Applying the cultural dimension of the CCA reveals how culturally patterned silences and moral framings influence both public discourse and professional behavior.

Vaccination is not openly rejected but rendered uncertain through hesitation, fragmented guidance, and limited dialogue. These dynamics are shaped by discomfort around adolescent sexuality, ambiguous professional roles, and weak institutional trust.

Efforts to increase vaccine acceptance must be grounded in trust, cultural sensitivity, and inclusive dialogue. Beyond technical solutions, strategies must address the social logics that govern what is said, how authority is communicated, and whose voices are legitimized. Without confronting these communicative patterns, resistance will likely persist despite scientific consensus.

In light of the observed gaps in guidance and messaging, future policy efforts should consider the development of a unified national communication protocol for healthcare professionals engaging with the public on HPV vaccination.

Breaking entrenched silences and stigma requires participatory approaches that empower both institutions and communities to co-create culturally resonant messages and reshape prevailing narratives around HPV vaccination.

## References

[1] Abi Jaoude J, Saad H, Farha L (2019). Barriers, attitudes and clinical approach of Lebanese physicians towards HPV vaccination; A cross-sectional study. Asian Pac J Cancer Prev.

[2] El Khoury J, Halabi R, Hleyhel M, El Rahman Kishly W, El Khoury R, Saleh N (2023). HPV vaccination prevalence among Lebanese female university students: A cross-sectional study. J Environ Public Health.

[3] Haddad SF, Kerbage A, Eid R, Kourie HR (2022). Awareness about the human papillomavirus (HPV) and HPV vaccine among medical students in Lebanon. J Med Virol.

[4] Hakimi S, Lami F, Allahqoli L, Alkatout I (2023). Barriers to the HPV vaccination program in the Eastern Mediterranean region: A narrative review. J Turk Ger Gynecol Assoc.

[5] Elissa N, Charbel H, Marly A, Ingrid N, Nadine S, Rachel A (2024). Knowledge and perception of HPV vaccination among Lebanese mothers of children between nine and 17 years old. Reprod Health.

[6] Hourani L, Zaatar M, Hoballah J (2024). Overview of knowledge, attitudes and barriers associated with HPV vaccination in Beirut, Lebanon. Glob Public Health.

[7] Zakhour R, Tamim H, Faytrouni F, Makki M, Hojeij R, Charafeddine L (2023). Determinants of human papillomavirus vaccine hesitancy among Lebanese parents. PLoS One.

[8] Abou El-Ola MJ, Rajab MA, Abdallah DI (2018). Low rate of human papillomavirus vaccination among schoolgirls in Lebanon: Barriers to vaccination with a focus on mothers' knowledge about available vaccines. Ther Clin Risk Manag.

[9] Yacoubian AA (2016). Perceptions of Lebanese Female University Students About the Human Papillomavirus (HPV) Vaccine: A Qualitative Study. Perceptions of Lebanese female university students about the human papillomavirus (HPV) vaccine : a qualitative study.

[10] Dutta MJ (2007). Communicating Health: A Culture-Centered Approach. https://www.politybooks.com/bookdetail?book_slug=communicating-health-a-culture-centered-approach--9780745634913.

[11] Dutta MJ (2022). Culture-centered approach to communicating health and development: Communication, social justice, and social change. The Handbook of Global Interventions in Communication Theory.

[12] Braun V, Clarke V (2006). Using thematic analysis in psychology. Qual Res Psychol.

[13] Airhihenbuwa CO, Dutta MJ (2012). New perspectives on global health communication: Affirming spaces for rights, equity, and voices. The Handbook of Global Health Communication.

[14] Xu MA, Choi J, Capasso A, DiClemente RJ (2024). Improving HPV vaccination uptake among adolescents in low resource settings: Sociocultural and socioeconomic barriers and facilitators. Adolesc Health Med Ther.

[15] Gulle BT, Kiran P, Celik SG, Varol ZS, Siyve N, Emecen AN, Duzel H (2024). Awareness and acceptance of human papillomavirus vaccine in the Middle East: A systematic review, meta-analysis, and meta-regression of 159 studies. Epidemiol Infect.

[16] Wiot F, Shirley J, Prugnola A, Di Pasquale A, Philip R (2019). Challenges facing vaccinators in the 21(st) century: Results from a focus group qualitative study. Hum Vaccin Immunother.

[17] Pavlovic D, Sahoo P, Larson HJ, Karafillakis E (2022). Factors influencing healthcare professionals' confidence in vaccination in Europe: A literature review. Hum Vaccin Immunother.

[18] Ozawa S, Stack ML (2013). Public trust and vaccine acceptance--International perspectives. Hum Vaccin Immunother.

[19] Ayodele JO, Kromberg Underwood ML, Al Ammari D, Goldstone K, Agogo E (2024). Enhancing trust and transparency for public health programs. Modernizing Global Health Security to Prevent, Detect, and Respond.

[20] Dutta MJ, Thaker J (2019). ‘Communication sovereignty’ as resistance: Strategies adopted by women farmers amid the agrarian crisis in India. J Appl Commun Res.

